# How Will the Future of Work Shape the OSH Professional of the Future? A Workshop Summary

**DOI:** 10.3390/ijerph17197154

**Published:** 2020-09-30

**Authors:** Sarah A. Felknor, Jessica M. K. Streit, L. Casey Chosewood, Michelle McDaniel, Paul A. Schulte, George L. Delclos, on behalf of the Workshop Presenters and Participants

**Affiliations:** 1 National Institute for Occupational Safety and Health, Atlanta, GA 30333, USA; LChosewood@cdc.gov; 2 National Institute for Occupational Safety and Health, Cincinnati, OH 45226, USA; JStreit@cdc.gov (J.M.K.S.); PSchulte@cdc.gov (P.A.S.); 3 Southwest Center for Occupational and Environmental Health, The University of Texas Health Science Center at Houston School of Public Health, Houston, TX 77030, USA; Michelle.R.McDaniel@uth.tmc.edu (M.M.); George.Delclos@uth.tmc.edu (G.L.D.)

**Keywords:** expanding occupational safety and health paradigm, future of work, occupational safety and health professional, training and education, Total Worker Health^®^

## Abstract

Rapid and profound changes anticipated in the future of work will have significant implications for the education and training of occupational safety and health (OSH) professionals and the workforce. As the nature of the workplace, work, and the workforce change, the OSH field must expand its focus to include existing and new hazards (some yet unknown), consider how to protect the health and well-being of a diverse workforce, and understand and mitigate the safety implications of new work arrangements. Preparing for these changes is critical to developing proactive systems that can protect workers, prevent injury and illness, and promote worker well-being. An in-person workshop held on February 3–4, 2020 at The University of Texas Health Science Center (UTHealth) School of Public Health in Houston, Texas, USA, examined some of the challenges and opportunities OSH education will face in both academic and industry settings. The onslaught of the COVID-19 global pandemic reached the United States one month after this workshop and greatly accelerated the pace of change. This article summarizes presentations from national experts and thought leaders across the spectrum of OSH and professionals in the fields of strategic foresight, systems thinking, and industry, and provides recommendations for the field.

## 1. Introduction

The world is undergoing major changes in the way work is performed, the workforce, and the workplace. With the goal of increasing productivity and the greater incorporation of technology, the pace of work has intensified. While short-term, temporary employment arrangements represent greater flexibility for employers, they can translate into more precarious situations for workers; lower pay for equivalent education, skills, and experience compared to those with long-term contracts; fewer benefits; and greater turnover [1,2,3]. Thirty percent of the U.S. workforce now engages in nonstandard work arrangements, such as contingent work, temporary contracts, and part-time work [4]. Additionally, estimates of teleworking under the COVID-19 pandemic reached upwards of 50% of all employed U.S. adults, and that number is expected to increase long after the pandemic [5]. Future of work scenarios describe an increasing global reliance on the informal sector and hazardous work exposures that are exacerbated by work-life stress and health consequences of precarious work [6].

Factors influencing worker health and well-being now go beyond traditional occupational safety and health (OSH) hazardous exposures and include changing demographic profiles (e.g., aging workers), greater burden of chronic disease, varying employment arrangements including informal work with little to no protections, shifts in work organization, increased psychosocial stressors, and the role of technology and related intensification of work demands. These combine with individual health and lifestyle and factors in the home, community, and general society to affect worker health and well-being [7]. Recent years have also seen a strong movement toward measuring worker well-being as a major safety and health outcome [7,8]. Together, these changes underscore the need for an expanded focus for OSH that goes beyond simply summing workplace illness and injury prevention with health promotion [9]. This expanded focus can significantly transform how we train future OSH professionals, conduct OSH research, and design forward-thinking policies to maximize worker health and well-being. The history of OSH has been one of adapting to change and an expanded focus can create the roadmap for OSH professionals of the future.

In 2019, the UTHealth School of Public Health Southwest Center for Occupational and Environmental Health entered into a three-year Cooperative Agreement with the U.S. National Institute for Occupational Safety and Health (NIOSH; Grant #U13OH011870) to advance this paradigm shift in focus for OSH. This cooperation seeks to contribute to the public discourse through a series of dissemination activities that bring together thought leaders, researchers, professionals, and practitioners representing a broad, interprofessional audience focusing on the OSH training, research, and policy/application needs in the future of work. The first year’s activities include a series of topic-specific workshops that center on examining workforce changes and how they will require refocusing OSH training and research. These workshops are preparatory to a three-day international, interprofessional conference, to be held in Houston, TX, USA, in early September 2021. The conference will be structured along the three areas of training, research, and policy/application; paying particular attention to how they interface with work, workers, and the workplace in the context of an expanded focus for OSH. Here, we report on the first of these workshops, held 3–4 February 2020, in Houston, TX, USA, at the UTHealth Cooley Conference Center.

## 2. Materials and Methods

This first workshop examined how the future of work will likely shape education and training for the next generation of OSH professionals. The objectives were to (a) examine the impact of future of work (FOW) on how we train the OSH professional workforce; (b) identify gaps and needs related to training and education; and (c) inform the agenda of the 2021 international conference. Workshop attendees represented a broad cross-section of stakeholders, including adult educators, worker representatives, government employers, industry professionals, the academic community, and consultants. Participants were identified using a modified snowball technique. The organizing committee generated an initial list of experts who were asked to nominate additional participants from a wide range of disciplines and professions relevant to the topic of the workshop. A final list of invited participants included experts from public, private, and non-governmental agencies representing the following sectors: academic research, education, construction, government, healthcare, management, organized labor, and workers’ rights. Most of the participants were from academic institutions (48%), followed by government (21%), industry (15%), non-governmental agencies (6%), and labor (4%).

Internationally recognized NIOSH thought leaders first provided an overview of the FOW and its likely impacts on worker health and well-being. Thereafter, the workshop format was structured along three themes: (1) innovative approaches to adult education, (2) the role of systems thinking in OSH education and training, and (3) what the future OSH professional “should” or “will” look like. Keynote speakers introduced each of these three themes and then facilitated small group discussions where participants addressed specific questions or challenges posed by the speakers. To foster greater interprofessional interaction, the small group composition was randomized and changed for each breakout session so that, by the end of the workshop, participants had worked with virtually all other attendees. Debriefing sessions provided an opportunity to link the discussions back to the workshop objectives, summarize gaps and needs, and generate conclusions. Workshop notes and recordings were transcribed and edited by the authors. Thematic analysis was carried out on small group discussion transcriptions using an inductive multi-phased approach to synthesize input and identify axial themes and representative statements. The results of the thematic analysis are shown in Table 1, Table 2 and Table 3.

## 3. Results and Discussion

### 3.1. The Future of Work and Implications for Occupational Safety and Health (OSH)

This section provides a summary of all workshop presentations and the results of group discussions that identified the challenges, gaps and needs of the three workshop themes described above.

#### 3.1.1. The NIOSH Future of Work Initiative and the Total Worker Health^®^ Approach

The NIOSH FOW Initiative was launched in 2019 and applies the Total Worker Health^®^ (TWH) framework by encouraging collaboration across organizational policies, programs, and practices. Central to both of these NIOSH futures-oriented priorities is the concept of worker well-being, which integrates the traditional OSH goal of protecting workers from occupational hazards with the promotion of health and illness prevention in the workplace and is being operationalized by NIOSH through its TWH program [10,11]. TWH promotes using more holistic approaches to broaden the focus from one narrowly centered on workplaces to those which incorporate both work-related and non-work-related factors that impact worker well-being, either positively or negatively [7]. Therefore, according to this contemporary conceptual framework, worker well-being emphasizes quality of life and is driven by the relationship between individual worker health and factors both at and outside the workplace, in order to have workers thrive and achieve their full potential [7].

Since the start of NIOSH TWH activities (https://www.cdc.gov/NIOSH/twh/) in 2011, there has been progress in advancing this NIOSH priority. Examples include funding and establishing TWH Centers of Excellence, improving the definitions and conceptual frameworks for well-being (noted in the previous paragraph), and identifying gaps and needs in research and applied interventions [7,10,12,13]. However, as knowledge advances, new needs and existing gaps emerge, and NIOSH seeks to build on these advances and identify current needs and gaps. NIOSH’s new FOW initiative (https://www.cdc.gov/niosh/topics/future-of-work/default.html) was launched to compile what is known about FOW scenarios and emerging trends and support new research, with an eye towards being able to forecast and anticipate risks that FOW may bring. Priority areas of focus include organizational design, changes in work arrangements, emerging technological demands (including job displacement), artificial intelligence, robotics, and other innovative technologies. The effort will highlight demonstration projects aimed at enhancing skills and economic security [14].

#### 3.1.2. Artificial Intelligence (AI) and Worker Safety

The unprecedented expansion of the use of AI in the workplace and its potential impacts on worker safety will change tasks workers perform and how they are protected from new and existing workplace hazards. AI will bring expanded use of sensors to detect and mitigate exposures, increased risks of human-robot interaction and autonomous vehicles, anticipated technological displacement, and greater incorporation of the internet of things into our lives. The large amount of data generated through technological advances will result in a greater need for occupational analytics and decision-making by decision data scientists and AI systems. AI will pose new challenges for OSH professionals as they prepare to respond technically and ethically to these changes [15].

#### 3.1.3. Towards an Expanded Focus for Occupational Safety and Health

**Figure 1:**
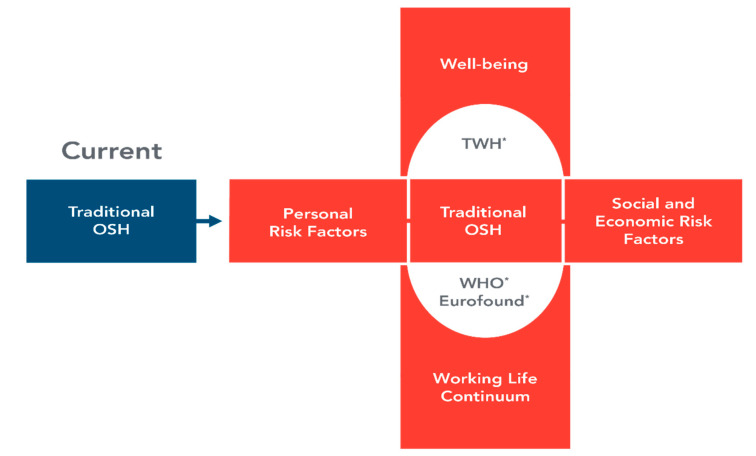
An expanded focus for occupational safety and health. * Horizontal and vertical expansion build on the work of WHO [20], Eurofound [21], and TWH [22,23].

The OSH field will need an expanded, more holistic focus to address challenges and changes posed by FOW scenarios to prepare the professionals of the future. This paradigm shift challenges traditional OSH systems by focusing on worker well-being as an outcome, goes beyond the prevention of workplace injury and illness or health promotion, and expands the types of hazards typically considered in the traditional OSH paradigm. The World Health Organization (WHO) Model for Action, various European efforts at well-being, and the NIOSH TWH Program provide important foundations for addressing changes in the world of work [9]. Beyond this, though, we need a more expansive paradigm to include greater recognition of both individual worker and workforce well-being as important OSH outcomes. Embracing this paradigm shift mandates a more expansive, systems thinking approach to better integrate traditional OSH with personal and socioeconomic risk factors, both horizontally (broadening the range of factors to examine their impact on health) and vertically (from a short-term, single job perspective to a work life continuum perspective encompassed by the overarching concept of well-being) [9]. This will require greater interprofessionalism, collaborative organizational leadership, proactive company policies, accountability, training, and engagement of management and employees, as well as following benchmarks over time and identifying opportunities for early corrective or enhancing interventions [13]. Moreover, as the paradigm expands, there will be a need for greater integration of systems thinking and transdisciplinary efforts, and for finding innovative ways to attract and train students into OSH professions. Systems thinking is the process of understanding the interconnection of elements (systems) that are organized to achieve a specific purpose [16]. Transdisciplinary efforts are those that cross multiple disciplines and professions and result in a broader and more holistic approach to problems solving strategies [17]. It is therefore likely that there will be a need for new disciplines and specialties in OSH or, at a minimum, a broader skill set and expanded training of traditional OSH professions to include occupational health psychology, human resource management, and TWH [18]. The model for this expanded focus for OSH was modified from Schulte et al. [9,19] and is presented in Figure 1.

### 3.2. Innovative Approaches to Adult Education

The future of learning and education must consider three major shifts in adult education that are shaping how we meet the learning needs of an increasingly diverse workforce in the future. These include new types of learners, new ways of learning, and new things to learn.

Changes in the nature of work are presenting challenges to the educational institutions to adapt or face the consequences. The increasing diversity of the workforce is also changing education and learning needs. There is a shifting balance of power between students and institutions, with the former carrying an increasingly greater weight and demanding novel approaches to learning [24]. Student types are transitioning from a traditional sequential learning pathway (e.g., from high school straight to college) to working students who attend part-time; are often older; come from diverse backgrounds; and have a need to balance work, study, and home life. Over time, they may have accumulated bits and pieces of educational credits and work experiences from different places. They are also more attuned to “on-demand” education, at a pace that fits more of a “just-in-time” lifestyle. And there is a transition from traditional major-based college tracks to more personalized learning, where students focus primarily on a declared life mission and seek to combine their educational experiences with the purpose of fueling that mission [24,25]. The COVID-19 pandemic has abruptly changed the education paradigm in the United States to essentially an entirely online platform, and this learning delivery method can be expected to see increasing demand in the future.

New approaches to learning are needed to more effectively engage nontraditional working students. In response, learning offerings are diversifying and moving towards faster, cheaper alternatives. Examples include more online, virtual, and micro-courses; massive open online courses (MOOCs) and other open courseware; certificate (rather than traditional degree) programs; expanded opportunities for applied/hands-on training; and models that support lifelong learning [26]. An important consequence of these changes will be their impact on accreditation of education programs, which are vital to OSH professional training (e.g., who will certify that coursework is adequate or that competencies have been achieved?).

Finally, there are new things to learn, much of it driven by the digital transformation that characterizes the fourth Industrial Revolution, and the need for greater AI-human interfacing at work and augmentation of human skills with technology. New skills that will be valued include systems thinking, human creativity and innovation, cultural and technological literacy, data analysis, problem-solving, working from a transdisciplinary perspective, social networking, and dealing with uncertainty, among others. There is a strong case to be made that many of these skills should start to be acquired early in life (e.g., during the K-12 experience) and solidified thereafter during higher education [25].

It is important to note that the workshop was designed to identify challenges, gaps, and needs facing the future of education and training of OSH professionals and not necessarily to provide concrete answers or next steps in the development of curricula, credentialing of OSH professions, or evaluation of new training methods. Therefore, the following questions were used to frame discussion of the key changes that are needed in education and learning in the future: (1) How can we more effectively meet the education and learning needs of an increasingly diverse OSH workforce? (2) In what ways can we expand our learning offerings to more effectively engage future OSH professionals? (3) With the rapid pace of change, including the rise of intelligent software and machines, what content is important for future OSH professionals to learn?

#### 3.2.1. Challenges

The discussion groups identified major challenges facing education and learning for future OSH professionals, such as diversity, technology, evaluation, and the decline in the number of current OSH professionals. Challenges around diversity include the growing heterogeneity in the characteristics and needs of OSH trainees and workers. Rapidly evolving technology will challenge how OSH trainees learn and how training is delivered. Evaluation challenges include the ways in which we measure, assess, or certify learning and skills development. Moreover, the decline in the number of OSH professionals challenges the training of the next generation of OSH professionals and opportunities for mentoring and meaningful placement of graduates.

#### 3.2.2. Gaps and Needs

Participants then identified gaps and needs for OSH education, conceptualizing them as key changes and important implications for the future of OSH training. A summary of these small group discussions is provided in Table 1. First changes to recruitment, educational approaches, classroom power dynamics, resilience training, and credentialing will be needed to more effectively serve future OSH learners; however, these changes will have implications for OSH core competencies, the security and longevity of OSH training programs, and approaches to quality control in OSH education. Next, changes to training diversity and interpersonal connectedness will be needed to more effectively engage nontraditional OSH learners with new types of learning, and these changes will have implications for OSH advocacy, community-based learning, and the realities experienced by both educators and learners. Finally, curricula should expand to include important new content and foster the development of a transdisciplinary workforce. Examples of new training content include an ecological model for worker health, the causes and consequences of new technologies at work, interpersonal skills, and advanced data analytics. Such changes will have implications for OSH culture, OSH training evaluation systems, and OSH educator preparation programs.

### 3.3. Systems Thinking Approach

Systems thinking provides an approach that can be applied to better understand how employers and workers are responding, often in counterproductive ways, to the connected problems of global competition, technology disruption, and stress-related illness. A systems approach to better understanding organizations has long been advocated by social psychologists [27]. *System* refers to an interconnected set of elements coherently organized in a way that achieves something [16]. *Systems thinking*, then, is the ability to understand these interconnections in such a way as to achieve a desired purpose, with the goal of knowing more about the whole system. Conventional thinking typically assumes problems and causes are clearly connected; that others are to blame; that short-term, often multiple, interventions result in long-term success; and that individual components of a problem can be optimized. In contrast, systems thinking does not assume an obvious connection between problems and causes; understands that quick fixes may not improve (and, in fact, may worsen) matters over time because of unintended consequences; places a greater emphasis on understanding and improving relationships among the individual components of a problem; and seeks to focus on a few coordinated changes and leverage points, implemented over time to assure sustainability [28].

A systems thinking approach can be useful in addressing complex issues in OSH directly related to the FOW as well as current OSH issues that will carry forward. Because of this, the European Union has added systems thinking to the core competencies for the public health professional [29]. In the U.S., schools of public health are shifting the traditional public health education paradigm to emphasize both systems thinking and interprofessionalism, which is defined as working with professionals outside the disciplines of public health and closely linked to transdisciplinarity [30,31].

To better understand interrelationships relevant to OSH in FOW and as an initial step towards affecting change, the following systemic questions should be considered: (a) who are the stakeholders and how might they view the issue? (b) what changes in systems structure (e.g., policies, practices, power dynamics, perceptions or mental models, purpose) can be proposed to help organizations address the issue? (c) what might be the unintended consequences of these proposed changes?

These questions were considered in the context of three FOW challenges that will impact OSH: (1) technology disruption—innovations that have significantly altered the way consumers, industries, and businesses operate; (2) global competition—competing organizations serving international customers; and (3) changing worker demographics—shifts in historic worker characteristics.

#### 3.3.1. Challenges

Several key stakeholder groups were identified as potentially impacted by challenges facing OSH in the future in the areas of technology, globalization, and demographics. These stakeholder groups include employers; specific subgroups of workers such as immigrants and seasonal, older, and younger workers; unions; recruiters and other human resource professionals; the tech industry; unemployment agencies; policymakers; governmental agencies; share- and stockholders; academic institutions; consumers; and the general public.

Changing worker demographics challenge communication and training needs. Challenges identified by technological disruption are brought on by innovations that have significantly altered the way consumers, industries, or businesses operate. Global competition is challenged by competing organizations, a decline in unionization, cross-cultural issues including miscommunication and changing values, and disparities in health and equality across companies and populations. A summary of the small group discussions around proposed changes and possible unintended consequences for each current issue is provided in Table 2.

#### 3.3.2. Gaps and Needs

The following gaps and needs were identified related to integrating a systems thinking approach into training the next generation of OSH professionals. Systems thinking should be taught early on and as part of a core curriculum versus an on demand soft skill. It should be viewed as not only a purely cognitive skill but one that includes broader skill sets of facilitation, spiritual work, and emotional intelligence. Interdisciplinary leadership and guidance will be important. Public health tends to still look at cause and effect linearly, while a systems approach would encourage a big picture view that understands other perspectives. Strong problem-solving skills will be needed to anticipate and adapt as change happens.

### 3.4. Profile of the OSH Professional of the Future

Issues related to the profile of the future OSH professional were presented from an employer perspective, with particular emphasis on environmental health sciences in business, the realities of a more distributed and mobile workforce, and the need for alignment with non-OSH professions.

Recognition of the need to address environmental health issues on a global scale is increasing within the business world [32]. Climate-related changes, such as extreme weather events, can have simultaneous effects on business operations and the surrounding community, creating a mutual dependence and responsibility for coordinated responses. Company statements of purpose are now more likely to go beyond simply assuring returns to shareholders, incorporating commitments to stewardship, global sustainability, and duties to community [33]. Workforces are more distributed, oftentimes around the world, and increasingly mobile. The increase in the use of short-term contracts results in workers having an increased number of jobs over a lifetime, at times coupled with several changes in career paths. This raises important questions for companies in terms of retaining critical skills and institutional memory [34]. Potential solutions include hiring based more on desired skill sets than educational background and combining subject matter experts with an empowered workforce. Additionally, the lines between work, home, and community are evermore blurred; and there is evidence that good health—including mental health—and happiness are drivers of productivity [35,36].

#### 3.4.1. Challenges

A significant challenge facing the OSH field is the risk of being marginalized if it cannot embrace and adapt to FOW, including how to deal with uncertainty. There are opportunities, but they will likely require important changes in how we educate the OSH professionals. One important question for the OSH community to consider includes how to best integrate the need for training in specific skills in OSH and allied disciplines with the need for training in “softer” skills, including leadership, corporate culture, and well-being.

Additional challenges include how to manage the transactional/gig nature of the new workforce. How do we protect the institutional knowledge when people are working shorter periods in any one company? With shorter tenure and more rapid turnover, it becomes increasingly challenging to find ways to retain knowledge and transfer it to a new and changing workforce. Another challenge that is not new to OSH is developing strategies for how to “sell” or promote the value of OSH to non-OSH audiences. The attributes OSH professionals of the future will need in order to combat these challenges and the strategies to foster development of such attributes are summarized in Table 3.

#### 3.4.2. Gaps and Needs

There is a need to incorporate multiple perspectives of different professions into OSH training and integrate different disciplines to create a transdisciplinary approach to problem solving. OSH professionals need greater interpersonal skills to help communicate up and down the line as well as translate across professions and stakeholder groups. The OSH profession needs a balance of topical specialists and broadly trained health and safety generalists. All OSH professionals need increased opportunities for cross-training and soft skills development. Additionally, there is a need for greater problem recognition and problem-solving skills in OSH that are transdisciplinary and anticipates new risks in the FOW.

## 4. Conclusions and Recommendations

The OSH professional of the future needs to take a more holistic approach that brings several opportunities to engage leadership in the development of company/agency statements of purpose that goes beyond shareholders. Interacting with finance and insurance systems will be necessary to support a healthy workforce. OSH should pay attention to and anticipate new risks posed by different FOW challenges. How the field responds to these challenges can help address the gradual marginalization of OSH by creating a proactive rather than responsive profile.

Academic OSH programs should develop new approaches and methods, creating opportunities for targeted and focused training that can be personalized. Central to this is using a transdisciplinary perspective to incorporate multiple disciplines, professions, and technology into OSH academic training. An area of curriculum that is missing from many mainstream OSH training programs is the health and safety of the informal sector. These workers labor under precarious conditions with non-traditional exposures that are not well characterized nor understood. As reliance on this sector increases, the need for and expanded focus for OSH to address this important part of the labor market increases as well.

Training programs should also integrate OSH practice earlier in the degree pathway and re-engineer competency-based learning to achieve personalized learning objectives. Developing modular or standardized training units that “fit” together as needed based on a menu-driven curriculum could serve to support both learner-centered specialty and core competencies without necessarily being based on the traditional formal degree pathway. For this to be successful, however, we must value and accept learning that occurs outside traditional academic degree programs and have a mechanism by which to evaluate and certify learning obtained with this approach. Finally, regardless of the learning pathway, we must find ways to incorporate OSH tenets earlier into the education and career decision-making process.

Responding to shifts in historic worker characteristics will create opportunities to change human resource practices and selection practices. Unions and organized labor will need a more diverse representation of the changing worker demographics to continue to be a sustained voice for workers. Managing innovations that have significantly altered the way consumers, industries, or businesses operate will be critical in the FOW. New policies and practices will need to shift to accommodate increasing demand for flexible work arrangements, and research will be needed to fill knowledge gaps through a collaborative effort that creates a shared understanding of what motivates a given industry. Combatting the unintended consequences of global competition including a decline in union power, cultural issues that result in changing values in work and life, and disparate health and equality between companies and people will require new and more legal protections, financial support systems to fund education, expanded health and retirement benefits, and harmonization of work standards and work-life fit.

These recommendations will help develop a roadmap toward an expanded focus for OSH, built on the traditional OSH paradigm and the TWH framework, to anticipate future education and training needs. A new approach to training OSH professionals that anticipates changes the future of work will bring is a critical next step to developing systems that not only protect workers by preventing potential injury and illness but also promote worker well-being over the work-life continuum to optimize a productive and healthy life course.

The conclusions and recommendations presented in this paper are based on the work of a limited number of subject matter experts. A majority of participants were from U.S. academic institutions with existing OSH training programs, and their opinions may be influenced by existing academic paradigms that focus on OSH issues of workers in the U.S. A small number of participants were from non-governmental and workers’ rights organizations which may have underrepresented the OSH issues associated with workers in the informal sector. These limitations may be addressed in the planning international conference in 2021, which will be designed with inputs from this workshop.

## Figures and Tables

**Table 1:** Key changes in education and learning *.

**New Types of Learners:**	
***How Can We More Effectively Meet the Education and Learning Needs of an Increasingly Diverse OSH Workforce?***	
Key Changes	Important and Provocative Implications for OSH
– Recruit from diverse backgrounds (experiences and demographics) – Evolve and adapt systems and approaches to align with learners’ diverse needs and preferences – Empower learners to take active responsibility for their own (virtual) learning – Teach students to deal with uncertainty and offer services to help them keep pace with rapid changes – Establish new systems that recognize on-the-job training and assess competencies and skills required for work placement	– Core requirements should meet today’s needs and fill today’s gaps, but they must also undergo continuous review for relevance – OSH educators will become irrelevant if they refuse to change and meet learners’ needs and programs lacking successful outcomes (e.g., job placement) may disappear – Non-traditional credentials require valid, effective assessment, accreditation, and marketing to be accepted and respected by science and industry
**New Types of Learning:**	
***In What Ways Can We Expand Our Learning Offerings to More Effectively Engage Future OSH Professionals?***	
Key Changes	Important and Provocative Implications for OSH
– Expand dual degree offerings and provide menu-driven curricula to facilitate OSH specialization – *Learn to be nimble:* Implement flexible, modular, nontraditional learning and teaching modalities (e.g., digital and virtual platforms; lifelong learning) – Actively combat the loss of social interaction and teamwork that can come with nontraditional (e.g., online) learning	– Early and frequent exposure to OSH through problem-based learning and cooperative experiences may help establish OSH as an accepted norm – Working with and in communities increases the applicability and transferability of training – Integrating virtual and augmented reality into online learning experiences will create new experiences for teachers and learners
**New Things to Learn:**	
***With the Rapid Pace of Change, What Content is Important for Future OSH Professionals to learn?***	
Key Changes	Important and Provocative Implications for OSH
– Include digitalization, societal reliance on technology, and the human-technology interface as key OSH training topics – Teach from a biopsychosocial (rather than biomedical) model for OSH – Bring in multiple disciplines to create a transdisciplinary workforce – Provide instruction in organizational change and change management, and create opportunities to develop “soft skills” (e.g., social skills, communication skills, emotional intelligence) – Foster skills in a variety of data collection, management, analysis, and interpretation techniques	– Expanding OSH paradigms by integrating aspects of Total Worker Health^®^ (e.g., personal and societal risk factors, worker well-being) will create systems thinkers – Knowledge and skills that are not traditionally a part of OSH will require new evaluation metrics – Trainers must have the right credentials and skills sets to teach new OSH topics

* Key Changes and Implications reflect an integrated summary of input provided by workshop breakout groups.

**Table 2:** Systems approach to occupational safety and health (OSH) pressing issues *.

Challenge	Proposed Changes	Unintended Adverse Consequences
Technological disruption *Innovations that have significantly altered the way consumers, industries, or businesses operate*	– Build a shared understanding of tech disruption, antecedents and consequences of new tech adoption – Adopt practices and policies that support and empower workers (e.g., bottom-up decision making, flexible work, continuing education and skills building, job security, organized labor, regulations for workload or work time)	– Increased divide of high and low skilled jobs; reinforced and accelerated social divide; under/unemployment – Blurred work-life boundaries; reduced work hours and benefits; increased workload – Changes in competition (e.g., businesses closures); loss of profits, knowledge, and jobs
Global competition *Competing organizations serving international customers*	– Support organized labor and provide protections for whistleblowers and intellectual property – Develop systems that both recognize success and support those who struggle – Advocate for global standards for care, worker benefits, and OSH regulations – Foster personal and professional growth and well-being for workers	– Decline in unions and decline in trades; loss of union and workplace rights – Culture destruction; loss of cultural identity; cross-cultural miscommunication and misinformation – Increasing disparities at the organizational, corporate, and social-ecological levels
Changing worker demographics *Shifts in historic worker characteristics*	– Provide sufficient compensation and benefits (e.g., family care options, paid time off (vacation and sick), reasonable accommodations) – Diversify the workforce through inclusive hiring practices – Allocate funding to represent taxpayer needs and interests – Balance profits with corporate social responsibility programs, philanthropy, and volunteer efforts; and foster initiative-driven community-based partnerships – Train workers on OSH to meet a given workforce’s needs – Build mentorship capacity and provide access to goal-directed reskilling and upskilling opportunities – Provide transparent evaluations and continuous feedback at the worker and organization levels – Offer work flexibilities that enhance quality of life for workers and their families	– Wasted resources due to ‘fixes’ that fail to meet workers’ needs – Over- or underuse of benefits – Perceived favoritism of certain groups; nonverbal/implicit bias during hiring; increased discrimination – Disengagement; loss of confidence in management; waning loyalty to employer – miscommunications due to inadequate or excess communication; language barriers – Vacated positions due to reskilling and upskilling – Litigation; increased benefits packages – Blurred work-life boundaries

* Issues, Changes, and Consequences reflect an integrated summary of the input provided by workshop breakout groups.

**Table 3:** Profile of the OSH professional of the future *.

Attribute	Strategies to Foster Attribute Development
Systems thinking *Takes a holistic approach that views the interconnectedness of parts and function of the whole*	– Develop capacity to recognize work-related health problems and issues, especially for vulnerable groups (e.g., emergency responders, construction workers) Focus on holistic approaches to problem solving – Create opportunities to partner with and learn from other disciplines, such as public health, industrial hygiene, environmental health, geography, social services and mental health, and risk management (e.g., insurance experts and business strategists) – Increase attention on health and safety for recovery and rebuilding, including employers, OSH, public health, education, and special populations (e.g., immigrant workers and their communities, employees affected by public health emergencies)
Emotional intelligence *Maintains strong interpersonal skills (e.g., positive attitude, strong work ethic, clear communication*)	– Include leadership, marketing, and communication skills in OSH training programs – Expand OSH response capacity through cross-training, especially for mid-career professionals
Collaboration *Cooperates with other disciplines and professional fields*	– Identify a broad set of OSH collaborators, such as policymakers; professional societies and groups (e.g., ACOEM, ACGIH, ASA); employers (including small, medium, and large enterprises); city, county, and state governments; public health professionals; medicine and nursing professionals; environmental health and safety professionals; social scientists; and wellness/health promotion groups – Provide strategies to identify key conceptual overlaps with stakeholders and approaches for capitalizing on shared interests
OSH champion *Advocates for the OSH cause at all times and to all audiences*	– Embed OSH professionals into industry in order to bring recognition to the relevance and importance of OSH, build leadership support, raise employee awareness, and purposefully prioritize organizational problems – Encourage interaction between OSH and non-OSH professionals to engage wider group of professionals to advocate for and support frontline OSH activities. – Lead by example, leveraging major public health events (e.g., floods, pandemics) to highlight community-wide relevance of OSH.

* Attributes and strategies reflect an integrated summary of the input provided by workshop breakout groups.

## Appendix A. Workshop Speakers, Participants, and Organizing Committee Members

This appendix provides the names and affiliations of the workshop speakers, participants, and organizing committee.

**Workshop Speakers:**

Andy Hines, PhD (Human Development and Consumer Sciences, University of Houston, Houston, TX 77204, USA)

John Howard, MD, MPH, JD, LLM, MBA (Director, CDC NIOSH, Washington DC 20201, USA)

Paul A. Schulte, PhD (CDC NIOSH, Cincinnati, OH 45213, USA)

David P. Stroh, MA (Bridgeway Partners, Berkeley, CA 94703, USA)

Sara L. Tamers, PhD, MPH (CDC NIOSH, Washington DC 20201, USA),

Samuel Walker, PhD, MSPH (BP International, Houston, TX 77079, USA)

**Workshop Participants:**

Bethany Alcauter, MPH (Workers’ Defense Project, Austin, TX 78723, USA)

Ben Amick, PhD (Department of Epidemiology, University of Arkansas for Medical Sciences, Little Rock, AR 72205, USA)

Faiyaz Bhojani, MD, DrPH, FACP (Shell Health, Houston, TX 77002, USA)

L. Casey Chosewood, MD, MPH (CDC NIOSH, Atlanta, GA 30345, USA)

Houshang Darabi, PhD (Department of Mechanical and Industrial Engineering. University of Illinois at Chicago, Chicago, IL 60607, USA)

Annamaria Davidson, MD, MPH (Memorial Hermann Hospital at Memorial City Medical Center, Houston, TX 77024, USA)

Rena S. Day, MS, PhD (Department of Epidemiology, Human Genetics, and Environmental Sciences, UTHealth School of Public Health, Houston, TX 77030, USA)

Rosandra L. Daywalker, MD (UTHealth School of Public Health, Houston, TX 77030, USA)

Robert Emery, DrPH (UTHealth School of Public Health, Houston, TX 77030, USA)

Mike Flynn, MA (CDC NIOSH, Cincinnati, OH 45213, USA)

Thomas G. Grumbles, CIH (Board for Global EHS Credentialing, Cypress, TX 77433, USA)

Rebecca Guerin, PhD (CDC NIOSH, Cincinnati, OH 45213, USA)

Ayanna Jones, MD, MPH (Chevron Services Company, A Division of Chevron USA Inc., Houston, TX 77022, USA)

Walter Jones, MS, CIH (Laborers’ Health & Safety Fund of North America, Washington DC 20006, USA)

Linda Lee, DrPH (Linda D. Lee Healthcare Consultants, LLC, Austin, TX 78759, USA)

Silvia Maberti, PhD, CIH (Exxon Mobil Biomedical Sciences, Inc., Spring, TX 77389, USA)

Katherine Ann Murray-Del Aguila, CIH (BP Corporation, Houston, TX 77253, USA)

Dalia Nessim, MD, MPH (UT Health North Campus Tyler, Tyler, TX 75708, USA)

Rene Pana-Cryan, PhD (CDC NIOSH, Washington DC 20201, USA)

Trevor Peckham, MS, MPA (University of Washington, Seattle, WA 98195, USA)

Francisco Perez, PhD (UTHealth School of Public Health, Houston, TX 77030, USA)

William B. Perkison MD, MPH, FACOEM (UTHealth School of Public Health, Houston, TX 77030, USA)

Preethi Pratap, PhD, MSc (University of Illinois at Chicago, Chicago, IL 60607, USA)

Richard Rinehart, ScD (CPWR The Center for Construction Research and Training, Silver Spring, MD 20910, USA)

William Shaw, PhD (UConn Health, Farmington, CT 06030, USA)

James Shelton, MS (Houston North OSHA Area Office, Houston, TX 77304, USA)

R. Ronald Sokol, CSP (Contractors Safety Council of Texas City, Texas City, TX 77590, USA)

Jessica Streit, PhD, CHES (CDC NIOSH, Cincinnati, OH 45213, USA)

Naomi Swanson, PhD (CDC NIOSH, Cincinnati, OH 45213, USA)

Gregory Wagner, MD (Harvard T.H. Chan School of Public Health, Boston, MA 02115, USA)

Judy Wendt, PhD (Shell Health Americas, Houston, TX 77002, USA)

Lawrence Whitehead, PhD (UTHealth School of Public Health, Houston, TX 77030, USA)

**Workshop Organizing Committee *(italics*) and Staff:**

*George Delclos, MD, MPH, PhD (UTHealth School of Public Health)*

*Sarah Felknor, MS, DrPH (CDC NIOSH)*

*Paul Schulte, PhD (CDC NIOSH)*

*Michelle McDaniel, BS, CHES (UTHealth School of Public Health)*

Annette Allett (UTHealth School of Public Health)

Ritesh Mehta, MD (UTHealth School of Public Health)

Leah Merrill, BBA (UTHealth School of Public Health)

Manal Shafik, MEd (UTHealth School of Public Health)

Michael Silva, MSc (Universitat Pompeu Fabra)

Itunu Sokale, MBChB, MPH (UTHealth School of Public Health)

Susan Wu, MPH (UTHealth School of Public Health)
